# Could Elastography Be Used in the Prediction of Morbidly Adherent Placentation?

**DOI:** 10.1155/2016/4909431

**Published:** 2016-11-14

**Authors:** Stacey Davie, Wei Ling Wong, Teresa Clapham, Donald Angstetra, Rajit Narayan

**Affiliations:** ^1^Obstetrics and Gynaecology, Gold Coast University Hospital, Southport, QLD, Australia; ^2^Maternal Fetal Medicine Unit, Gold Coast University Hospital, Southport, QLD, Australia

## Abstract

Morbidly adherent placentation (MAP) is a condition in which the placenta is abnormally attached to the uterine myometrium. MAP is a complication of pregnancy that can cause significant morbidity to the mother and fetus and therefore early diagnosis is crucial in its management and prevention of adverse outcomes. Ultrasonography remains the primary diagnostic tool for MAP, with magnetic resonance imaging (MRI) serving as a secondary diagnostic modality. Elastography is a relatively new concept in ultrasound based imaging, which has found application in several fields of medicine, including obstetrics, primarily for evaluation of the firmness of cervical tissue in a preterm labour setting. We report a case on a patient who was diagnosed with placenta increta on ultrasound, aided by elastography and her subsequent management with an en bloc hysterectomy.

## 1. Case Report

This 31-year-old P0323 was 12 weeks into her sixth pregnancy at the time of initial referral to the Maternal Fetal Medicine Unit at the Gold Coast University Hospital, for planning of antenatal management, given her past obstetric history. Each of her previous three confinements resulted from preterm premature rupture of membranes and ensuing preterm labour between 27 and 28 weeks of gestation. She had to be delivered by Caesarean section in her last two confinements (fetal distress and elective repeat, resp.). She had retained placenta following her last Caesarean section, for which two separate hysteroscopy-guided curettage procedures had to be performed, placental histology confirming placenta accreta.

**Figure 1:**
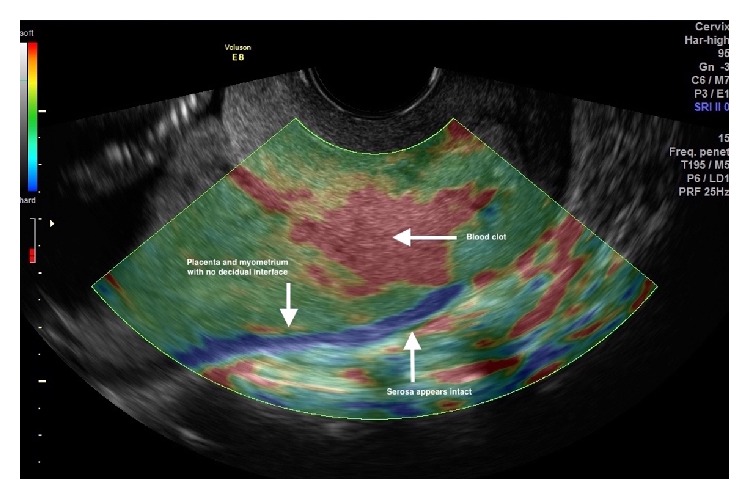
Elastogram demonstrating placental invasion into myometrium (placenta increta) with an intact uterine serosa.

**Figure 2:**
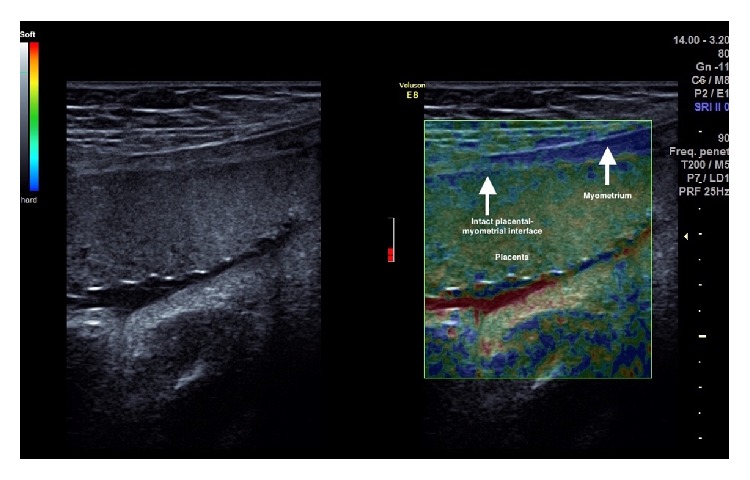
Elastogram demonstrating normal placenta-myometrium interface.

**Figure 3:**
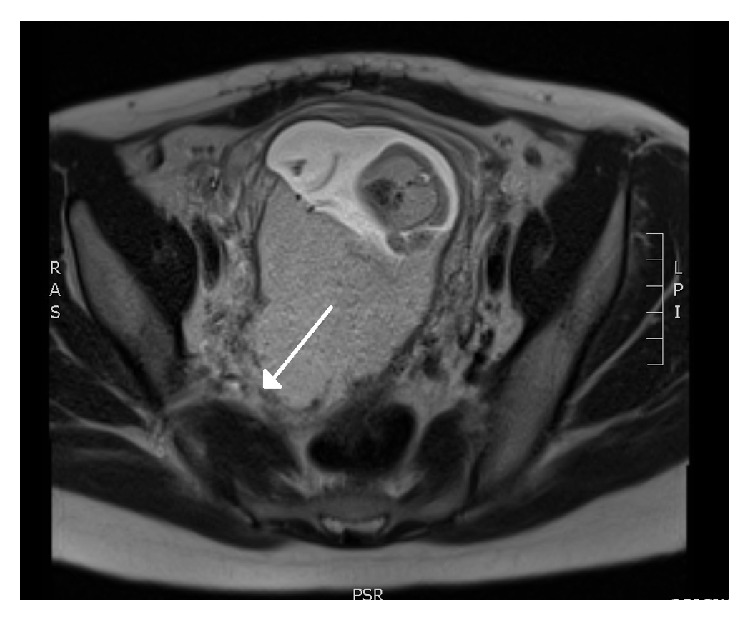
MRI demonstrating placental invasion into the myometrium (arrow).

At the initial ultrasound assessment using the Voluson E8 (GE Healthcare) [1], the fetus appeared morphologically normal. A transvaginal ultrasound with a RIC5-9-D transducer demonstrated a shortened cervix, 23 mm in closed length. A complete posterior placenta previa was recognized. The posterior decidual interface was not clearly visualized and turbulent blood flow was visualized at the interface, further raising suspicion about abnormal invasiveness of the placenta. An elastogram was performed at the same time [1]. Subjective interpretation of the elastogram was that this was a case of morbidly adherent placentation (MAP) in the posterior uterine wall, not to the extent of percreta. This was based on lack of a distinct zone of elasticity interspersed between the placental zone and the myometrial zone, which measured 2 mm in thickness. The uterine serosa appeared intact along its entire length on elastogram (Figures 1 and 2). An urgent MRI was arranged soon after this first visit, which confirmed MAP based on regional discontinuity of the uteroplacental interface (Figure 3).

**Figure 4:**
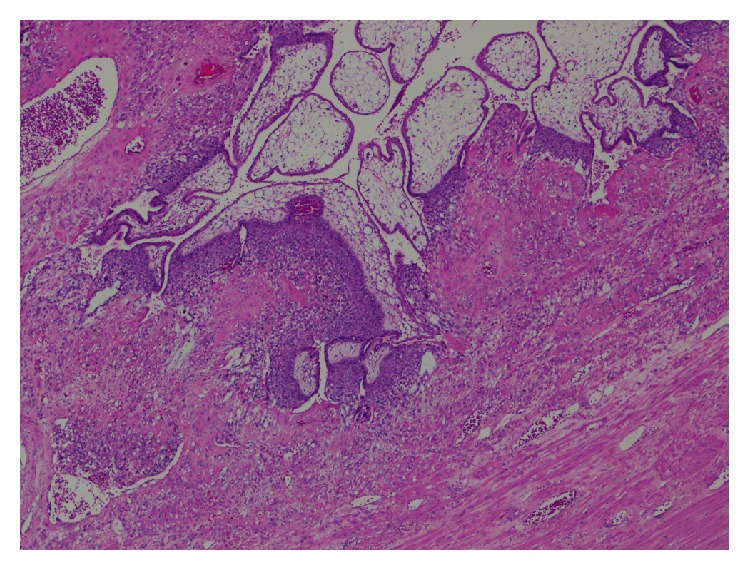
Histopathology: placenta increta with myometrial fibers immediately apposed to placental villi in the absence of intervening decidua.

Despite being on vaginal progesterone supplementation, the cervix continued to efface and two weeks from the initial assessment, the patient presented with heavy vaginal bleeding. On ultrasound assessment the cervix was almost fully effaced. The vaginal bleeding continued to intensify, necessitating an emergency en bloc hysterectomy, that is, hysterectomy with fetus in situ. The procedure was straightforward, with a total blood loss at surgery of about 500 millilitres. The woman had an uncomplicated postoperative course and made good recovery. Subsequent histology demonstrated invasion of immature chorionic villi into the myometrium, confirming placenta increta (Figure 4).

## 2. Discussion

Morbidly adherent placenta (MAP) refers to an abnormality of placental implantation into the myometrium as a result of defective decidualization of the implantation site. Normally, chorionic villi attaches to the decidual interface, but in the instances where the decidua is absent, attachment occurs directly to the myometrium. MAP is classified based on the extent of placental invasion into the uterine myometrium. Adherence of chorionic villi to the basal decidua constitutes placenta accreta; when the villi invade into the myometrium it is classified as placenta increta; and in placenta percreta, the villi penetrate through the myometrium and into or beyond the uterine serosa. The overall incidence of MAP is approximately 1 in 2500 pregnancies of which placenta accreta accounts for 79 percent, placenta increta 14 percent, and placenta percreta 7 percent [2–4]. Risk factors for MAP include previous Caesarean sections, history of uterine surgery, and advanced maternal age [5, 6]. Prior Caesarean delivery is the most important risk factor for MAP. In the absence of placenta previa, the probability of MAP increases from 0.3 percent with one previous Caesarean section to 2.5 percent with three or more prior Caesarean sections [3, 7, 8]. In women with placenta previa, the frequency of MAP increases from 1 to 5 percent without prior Caesarean delivery to 50 to 67 percent with four or more prior sections [9]. MAP confers significant risk of morbidity and mortality to the mother and fetus alike. It may present as antepartum haemorrhage, necessitating preterm delivery of the fetus, or life threatening, profuse postpartum haemorrhage requiring hysterectomy and large volume blood transfusion [10]. Hence it is imperative that robust prenatal diagnostic tests be in place so as to maximise the chance of prenatal diagnosis. Early diagnosis allows more aggressive preoperative planning and management to minimize any potential adverse outcomes.

Ultrasonography, being readily available at reasonable cost, is routinely used as the first level of screening for MAP. Two-dimensional (2D) ultrasonography remains the cornerstone in the assessment of placenta location and implantation. Addition of colour Doppler improves detection rates. A recent meta-analysis looking at prenatal sonographic identification of invasive placentation using ultrasound reported sensitivity of 90.7 percent, specificity of 96.9 percent, positive likelihood ratio of 11, and negative likelihood ratio of 0.16 [11]. Ultrasonography is therefore a robust primary screening tool for MAP. The current secondary screening modality, namely, MRI, is expensive and not as readily accessible to the entire population geographically. MRI is however particularly useful in cases of posterior placenta or in women with high Body Mass Index (BMI). Although MRI has a high predictive accuracy in assessing both the depth and topography of placental invasion, there is no difference in either the sensitivity or the specificity between ultrasound and MRI for the detection of invasive placentation [12]. When used in conjunction with ultrasound, MRI rarely changes the surgical management of MAP [13]. Also, despite the high diagnostic accuracy of ultrasonography and MRI in the diagnosis of MAP, there remain some questions around the consistency and interobserver agreement of their interpretation.

Three-dimensional (3D) sonography with power Doppler may be a useful addition to our current diagnostic armamentarium [14]. With this case report, we propose the use of quantitative elastography to provide more objectivity in the diagnosis of MAP. The basic principle of elastography is to detect tissue strain by assessing the echo amplitudes of the subjective tissue when compressed and uncompressed. The stiffness (strain) of various tissues is shown by a different displacement of echoes. Soft tissue demonstrates a higher displacement or strain and low strain indicates a “harder” tissue. Despite the plethora of different elastography methods that have become available, they all aim to display contrast for or measure quantities related to the comparison of the strain of tissue. Elastography has been widely used in the assessment of the liver, breast, and prostate [15]. In obstetrics and gynaecology, elastography has been used to assess uterine fibroids, subchorionic hematoma, and cervical length in the cases of preterm labour [16–18]. It has also more recently been used to evaluate differences in placental function between normal and preeclamptic pregnancies and may be useful as an aid in predicting preeclampsia [19, 20].

The delineation of exact placental areas that may be morbidly adherent cannot possibly be achieved in the prenatal setting with currently available diagnostic tools. Hypothetically, the absence of a discrete decidual interface in MAP should be detectable and quantifiable on elastography. The aim of the novel use of elastography in this setting is to screen “at-risk” women in early gestation for any placental invasion. This will allow a more accurate, objective, and appropriately timed diagnosis of MAP, thus potentially preventing any devastating complications arising from delay or misdiagnosis.

Prospective research is needed to test this hypothesis and its clinical applicability. Specifically, larger quantitative studies of this technique are required to demonstrate the potential advantages of elastography in the prediction of MAP.
